# User‐Centred Design of Multi‐DoF Prosthetic Wrist Controllers: Performance and Preference in Daily Living Tasks

**DOI:** 10.1049/htl2.70092

**Published:** 2026-07-31

**Authors:** Deepti Bharadwaj, Ingyun Ahn, Saurabh S. Deshmukh, Jiyeon Kang

**Affiliations:** ^1^ Mechanical and Aerospace Engineering University at Buffalo New York USA; ^2^ AI Convergence Gwangju Institute of Science and Technology Gwangju Korea (the Republic of)

**Keywords:** IMU‐based control, prosthetic wrist control, sensor‐driven assistive interface, upper‐limb assistive technology

## Abstract

This study presents a practical, IMU‐based control framework for 2‐DoF prosthetic wrists, designed to explore the feasibility of supporting functional wrist control in daily tasks. The system employs movement synergies inherent to the upper limb and offers three controller configurations: pronation–supination (PS), radial‐ulnar deviation (RUD) and a combined PS‐RUD mode. Ten healthy participants, simulating upper‐limb amputees, performed four representative activities of daily living in a virtual environment, during which both kinematic performance metrics and user‐perceived effort were recorded. Task‐specific controllers led to significant reductions in compensatory limb movements, and overall, the combined PS‐RUD controller was preferred by users regardless of task completion time. Participants reported lower physical and cognitive effort, underscoring the intuitiveness and usability of the suggested controller. These preliminary findings suggest the potential of a user‐centred control strategy for prosthetic wrists that could reduce training burden and facilitate seamless integration into everyday functional use.

## Graphical abstract

This study evaluates an IMU‐based, synergy‐driven control framework for multi‐DoF prosthetic wrists, evaluated across daily living tasks in a virtual environment. Results show that task‐specific controllers reduced compensatory limb motions and effort, while a dual‐axis configuration was preferred in complex orientation tasks regardless of task completion time. The findings highlight a user‐centered strategy that improves intuitiveness, usability, and integration of prosthetic wrists into daily activities.

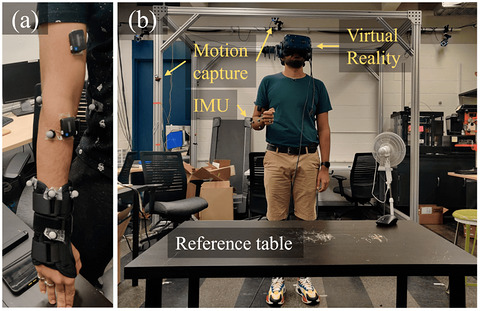

**Abbreviations**
PS: pronation–supination
RUD: radial‐ulnar deviation
RoM: range of motion
rANOVA: repeated‐measures analysis of variance
IMU: inertial measurement units
EMG: surface electromyography

## 1 Introduction

Upper‐limb prostheses aim to restore some autonomy and functional capability by replacing amputated limbs with artificial systems. Advancements in mechatronics and control technologies have enabled the development of high degrees of freedom (DoF) systems, including articulated hands, multi‐DoF prosthetic wrist modules and fully actuated prosthetic arms [1, 2, 3]. To control multi‐DoF prostheses, myoelectric prosthetic control has been explored for over half a century and is currently used in most of the actuated prosthetic systems [4]. Conventional myoelectric control operates an electric motor using sEMG signals to execute basic functions such as opening and closing a prosthetic gripper [5]. Early attempts to extend the myoelectric control to multi‐DoF prosthetic systems often relied on state machine architectures, where two sEMG signals were used to control a single joint, with joint switching enabled by co‐activation of the muscles [6]. However, these approaches lacked the ability to provide intuitive, simultaneous control across multiple joints, significantly limiting the prosthesis's dexterity and functional utility during daily tasks.

To address the limitations of state machine‐based control, pattern recognition techniques incorporating machine learning have been widely explored [6, 7, 8, 9]. However, their limited intuitiveness and the high cognitive effort required for reliable operation [10] have made it challenging to convert these methods from the experimental setting to everyday use. More recent studies have explored sequential deep learning models, such as LSTM, TCN and reinforcement learning techniques, to enable more natural and dexterous multi‐DoF prosthesis control [11]. Although these models have shown promising results in virtual or controlled environments, their translation to real‐world use remains challenging, particularly across varied activities of daily living (ADLs). Furthermore, sEMG‐based controllers are inherently sensitive to electrode shift, posture‐dependent muscle activation and transient changes due to muscle fatigue over extended use [12]. These persistent limitations have prompted growing interest in alternative control strategies that can offer greater robustness, intuitiveness and usability in everyday use.

As an alternative to sEMG‐based control methods, movement‐based techniques leverage inertial measurement unit (IMU) sensors to capture residual‐limb kinematics and actuate prostheses. These methods often exploit natural movement synergies, wherein proximal joint motions are used to control distal joints [13, 14]. For instance, movement synergy‐based controllers have mapped wrist pronation and supination to shoulder movements, enabling more intuitive and cognitively efficient prosthetic wrist control [13]. In this study, a similar synergy‐based control strategy is employed to coordinate proximal joint movements, thereby producing intuitive control inputs for distal wrist posture control. Unlike sEMG‐driven interfaces, movement‐based approaches rely on joint coordination patterns, offering an intuitive control paradigm with reduced training demands [13, 15]. Beyond traditional mathematical model‐based strategies, regression‐based frameworks have also been proposed to exploit these synergy‐based methods. Merad et al. [16] modelled elbow flexion and extension based on shoulder elbow coordination, while Peng et al. [17] extended this idea by predicting wrist rotation from shoulder and elbow angles using multivariate linear regression. These studies demonstrated that synergy‐driven control reduces compensatory movements and facilitates intuitive operation across both amputee and non‐amputee populations.

To expand these methodologies to multi‐DoF wrists, we previously proposed a novel synergy‐driven framework for controlling multi‐DoF prosthetic wrists using IMU signals [18]. This framework integrates a neural network classifier to infer task intent, specifically pronation/supination (PS) and radial/ulnar deviation (RUD) of the prosthetic wrist, followed by regression models that predict wrist angular velocities using proximal limb movement. Offline experiments demonstrated high classification accuracy and regression robustness, validating the feasibility of leveraging movement synergies for natural and intuitive multi‐DoF wrist control. In this study, we extend the framework into a real‐time setting to evaluate different prosthetic wrist configurations on kinematics, task time and user perception during daily activities. Ten participants were recruited to perform four ADLs with different wrist configurations. The evaluation is conducted in a physics‐based virtual environment using the MuJoCo engine.

## 2 Wrist Controller Algorithm

**FIGURE 1:**
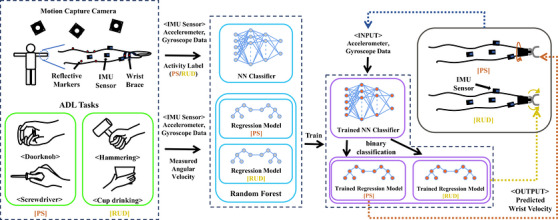
IMU‐based multi‐DoF prosthetic wrist control framework. ADL tasks are classified into PS or RUD using a neural network, and the corresponding regression model predicts wrist angular velocity of the prosthesis.

The multi‐DoF prosthetic wrist control framework, previously introduced in our earlier work [18], is depicted in Figure 1. It comprises two main components: a classifier that distinguishes between PS and RUD motions, followed by dedicated regression models for each identified motion type. Both the classifier and regression models were developed in Python 3.7.

### 2.1 Classifiers

A binary classifier was implemented the Keras 2.4.3 framework. It takes input signals from an IMU sensor and generates probabilities for the RUD and PS categories. The category with the higher probability is considered as the user's intended motion. The architecture comprises a densely connected neural network with two hidden layers, containing 10 and 5 neurons respectively, employing ReLU and Softmax activation functions. ReLU serves to process the input data nonlinearly, forwarding it to subsequent stages, whereas Softmax calculates the probability of each category, facilitating the selection of the most probable category. The loss function employed is binary cross‐entropy, with Adam optimizer used for optimization. The network configuration included the number of hidden layers and neurons, and was tuned by iterative testing in an attempt to prevent overfitting.

### 2.2 Regression Model

Regression models were created to predict the angular velocities of two primary movements, PS and RUD, using IMU data that was collected during ADL tasks. Scikit‐learn was used to create these models, and GridSearchCV was a useful tool for figuring out the most optimal tree structure. With 50 trees and a maximum depth of 40, the final Random Forest regression model performed remarkably well in predicting the angular velocities for both movements.

The implementation of the Random Forest regression model involves several detailed processes. Bootstrap sampling, the first step, involves randomly resampling the original dataset with replacement to create several sub‐datasets, each containing only a percentage of the original data, with some points appearing multiple times. This technique enhances diversity and reduces overfitting by ensuring that each decision tree learns from slightly different data. In the decision tree construction phase, each sub‐dataset is used to build its own decision tree, with a subset of all available features randomly selected at each node to find the optimal split. This approach improves the model's generalization capacity by allowing each tree to learn distinct features of the data. During aggregated prediction, the final prediction value for new data points is obtained by averaging the results from each tree, leading to more reliable and accurate predictions by minimizing the potential for individual tree errors. A neural network architecture with two hidden layers was used. The first layer had 10 neurons and the second layer had 5 neurons, and a small batch size of 8 was used to reduce overfitting. For further details on the model and its validation, please refer to our previous work [18]. The classifier was not retrained for this study when testing each individual, and its performance was characterized using accuracy, loss trajectories and validation results in [18]. Cross participant evaluation was performed using repeated leave out participant testing with F1 score, precision, recall and confusion matrices.

## 3 Methods

### 3.1 Participants

The study was approved by the University's Institutional Review Board (IRB STUDY00005631 Approval date: 9/2/2021). Participants provided written consent before the experiment. Participants included ten healthy individuals (2F/8M, 23.6 ± 2.1 years old), including three left‐handed participants. All participants provided their written informed consent prior to participation in this study. Potential participants were disqualified if they were under 18, had a history of neurological or orthopaedic conditions affecting their upper limbs, or exhibited significant self‐reported visual or auditory impairments that would interfere with their ability to follow the study protocol. Participants wore a wrist brace to remove active wrist motion. The brace was used to simulate the absence of wrist mobility in individuals with upper‐limb amputation.

### 3.2 Experimental Protocol

**FIGURE 2:**
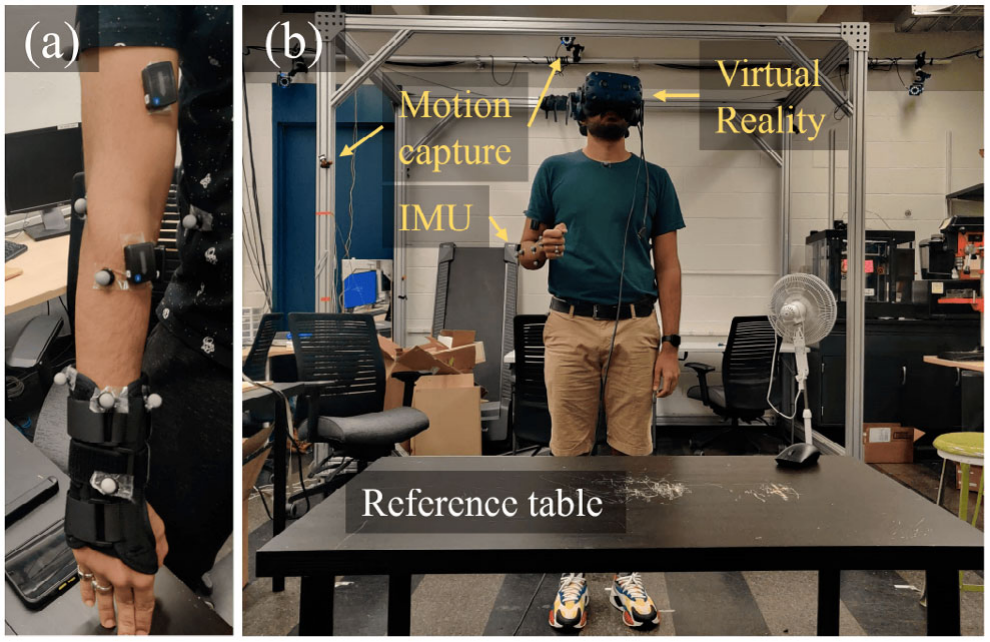
(a) Reflective marker and IMU placement. Participants emulated amputees by immobilizing the wrist. (b) Overall experimental set‐up.

**FIGURE 3:**
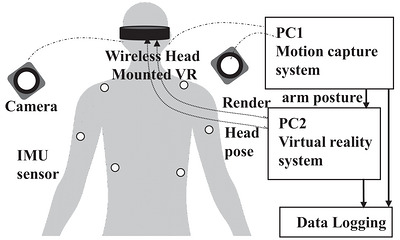
Setup of IMU sensors, motion capture markers, VR system and their communication between two PCs.

The experimental protocol was designed to enable within‐participant comparison among three prosthetic wrist controller configurations: PS, RUD and a combined dual‐axis controller. Ten motion capture cameras (Vicon, UK) were used to measure the upper limb kinematics of participants (Figure 2). For the multi‐DoF wrist control, IMU/sEMG sensors (Trigno, Delsys, USA) were attached to the participants' skin as shown in [18]. The IMU data were transferred to the framework and the desired wrist speed of the virtual multi‐DoF prosthetic wrist was output. Data from two sEMG sensors placed over the flexor carpi radialis (FCR) and extensor carpi radialis longus (ECRL) muscles on the forearm were used to control the virtual prosthetic gripper. Grasping and releasing motions were actuated by threshold‐based controls: higher FCR activity indicated grasping, while higher ECRL activity indicated releasing. Individualized threshold values were calibrated prior to the experiment. The sEMG threshold for gripper control was determined via repeated calibration trials to mitigate inter‐individual variability and maintain stable control throughout various sessions. The virtual reality (VR) environment was implemented using the MuJoCo physics engine and a VIVE Pro 2 head‐mounted display (HTC, Taiwan). Forearm motion capture data were streamed into the physics engine to synchronize the participant's actual forearm posture with that of the virtual prosthesis, as shown in Figure 3.

Each experiment followed a standardized procedure. Prior to the experiment, study team members showed participants tutorial video recordings of each activity and performed a live demonstration to ensure understanding of the task. After that, participants were made familiar with the VR environment and setup for about 5 min. Before starting the experiment, practice task trials were also provided until they felt comfortable conducting the experiment with the setup and the VR headsets, followed by randomization of task and controller order to reduce learning effects.

**FIGURE 4:**
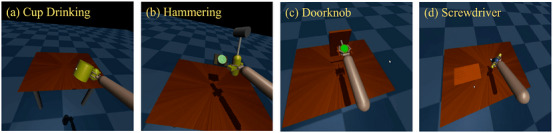
Four different ADL tasks in the virtual reality experimental setup.

**FIGURE 5:**
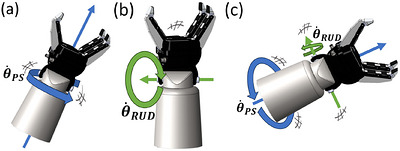
Three different types of wrist controllers' rotational directions: (a) PS controller, (b) RUD controller, (c) PS and RUD controller (combined PS‐RUD).

When the experiment started, participants were instructed to make a T‐pose, which is defined as positioning the upper arms at a 90‐degree angle relative to the torso, with elbows fully extended and aligned horizontally. Upon receiving a start signal, participants performed ten trials for each ADL task as shown in Figure 4. The four ADLs selected for this experiment are: drinking from a cup, hammering a nail, operating a doorknob, using a screwdriver. The four ADL tasks were selected based on clinical ADL assessments, specifically the AM‐ULA and the SHAP test, to represent common upper‐limb functional demands [19, 20]. The cup and hammering tasks were taken from the list of activities in the AM‐ULA assessment, which includes everyday actions such as drinking, lifting objects and using tools, while the doorknob and screwdriver tasks were taken from the SHAP test, which includes activities such as turning handles, rotating lids and manipulating utensils. The cup and hammering tasks involve radial and ulnar deviation in a primarily vertical motion, and the doorknob and screwdriver tasks produce continuous rotational motion through PS. For each task, completion was defined by a colour change of the virtual object from green to red, which occurred when the participant successfully reached the target position. The participants were asked to repeat each task 10 times. A 5‐min break was provided after completion the tasks. Three distinct controllers (Figure 5) and four experimental tasks were pseudo‐randomly assigned to the participants. For further details on the models and their validation, refer to our previous research [18]. The prosthetic hand was controlled using a threshold‐based decision algorithm, where the hand opened or closed depending on whether the input signal exceeded a predefined threshold value.

### 3.3 Data and Statistical Analysis

**FIGURE 6:**
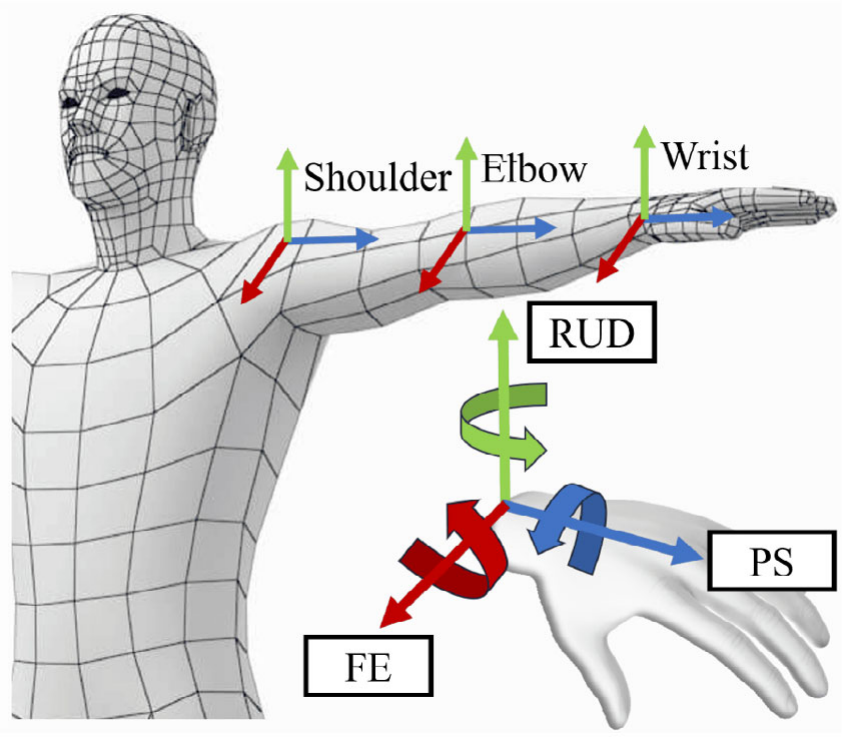
Definition of 3‐DOF rotational movements of the upper arm. *x*, *y*, *z* axes used for Euler angles.

To compute kinematics in the upper limb, the Euler angles were computed. The normal vectors to the planes defined by the three marker points on each body segment and the fixed frames were used to construct a rotation matrix. The Euler angles were determined using the rotation matrix. In Figure 6, the rotation about the *x*‐axis (yaw), the rotation about the *y*‐axis (roll) and the rotation about the *z*‐axis (pitch) were employed to depict Euler angles. The upper limb joint angles are represented by the three Euler angles with respect to the T‐pose. In this study, compensatory movement was defined as excessive shoulder and elbow motion beyond that required for nominal task execution when operating the prosthesis with each wrist controller configuration. Compensatory motion was quantified using joint angle range, and comparisons were performed across the three controller conditions. Physical and mental effort were assessed using the NASA Task Load Index (NASA‐TLX), a validated multidimensional instrument commonly used to quantify perceived workload in human‐machine interaction and rehabilitation studies [21].

The range of motion (RoM) from each individual data were combined to create group data. For both RoM and task completion time, a repeated‐measures analysis of variance (rANOVA) was used to analyse this batch of data and assess quantitative differences between the three controllers across four tasks. Sphericity was assessed using Mauchly's test, and if violated, Greenhouse–Geisser correction was applied. Bonferroni–Holm correction was applied for post hoc pairwise comparison of the controllers [22]. User‐preferred controller rankings were compared across controllers using the Friedman test. When significant, pairwise comparisons were performed using Wilcoxon signed‐rank tests. All statistical analyses were performed using SPSS 27 (IBM Corp., Armonk, N.Y., USA), with α=0.05.

## 4 Results and Discussion

### 4.1 Range of Motion and Task Completion Time

**FIGURE 7:**
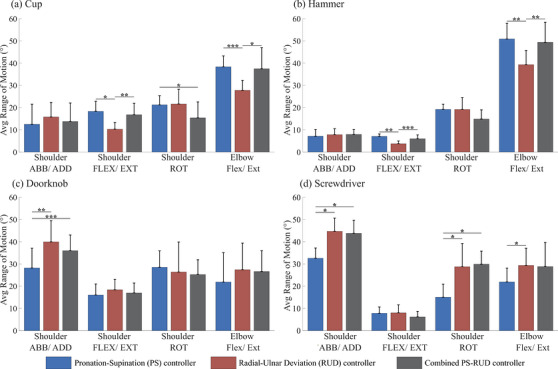
Upper arm range of motion for ADL tasks. Bars represent the average across the group. Error bars are one standard deviation. *P < 0.05, **P < 0.01, ***P < 0.001.

The group's data were compiled to compare the kinematic RoM of the upper arm. Figure 7 indicates the RoM of four different tasks. For the cup task, a significant difference was observed in the shoulder flexion/extension (*p* < 0.01) and elbow flexion/extension (*p* < 0.01) RoM across all three controllers. This is because the associated wrist movement (wrist RUD) for this task is correlated with the elevation of the object by shoulder flexion/extension. Comparison between the controllers showed that the RUD controller required 43.86% less shoulder flexion/extension than PS (*p* < 0.05) and 38.79% less than the combined PS‐RUD controller (*p* < 0.01) (see Figure 7a). The same trend was also observed for elbow flexion/extension, where RUD required 27.61% less elbow motion than PS (*p* < 0.001) and 25.97% less than combined PS‐RUD (*p* < 0.05). This is because the RUD controller is effective at generating the motion required for the cup task. In the controller without the wrist RUD motion, the user must compensate by performing additional angular movements in the elbow and shoulder along the direction of the wrist RUD motion to tilt the cup. Additionally, a notable difference was observed in shoulder rotation (*p* < 0.05), with a significant distinction particularly between the PS and combined PS‐RUD controller (*p* < 0.05), with the combined PS‐RUD controller requiring 10.28% less shoulder rotation than PS. This difference can be attributed to the compensatory shoulder rotational movements required when using single‐axis controllers (PS or RUD), compared with the combined PS‐RUD controller, which provides a more direct, and efficient movement path to the target by utilizing two DoFs of the wrist.

Similar to the cup task, for the hammering task, shoulder flexion/extension shows a significant difference (*p* < 0.001) in the RoM across the three controllers. The RUD controller significantly reduced the required RoM by 46.94% compared to PS (*p* < 0.01) and by 37.36% relative to combined PS‐RUD (*p* < 0.001) (Figure 7b). A significant difference (*p* < 0.01) is also observed in the RoM of the elbow between the controllers. The RUD controller significantly reduced the average RoM required to actuate the prosthetic wrist by 22.69% compared to PS (*p* < 0.01) and by 20.41% to combined PS‐RUD (p < 0.01). These findings are consistent with previous studies [15, 16], which also reported reduced compensatory upper‐limb movement when the active wrist DoF was matched to task‐specific demands. The RoM of the hammering task is different from that of the cup task because participants used their elbows more than their shoulders. An average of 15 degrees of shoulder flexion/extension for the cup task compared to 6 degrees of shoulder flexion/extension for the hammering task. It should be noted that the controller relies heavily on shoulder movements to predict wrist velocities, which may increase user effort to deliver the intent of the user. However, the results in Figure 7b, which demonstrate a significantly reduced mean RoM, suggest that the RUD controller minimizes compensatory movements more effectively than other controllers, despite the smaller range of shoulder motion. This indicates that, even for tasks that require less shoulder movement, the RUD controller may provide wrist‐velocity estimates that are consistent with the intended task direction under the tested conditions.

For the doorknob task, shoulder abduction/adduction shows a significant difference (*p* < 0.001) in the RoM across the three controllers. This is because the associated wrist movement (wrist PS) for this task is correlated with shoulder abduction/adduction, which rotates along the P/S axis. Comparison between controllers indicates that the PS controller significantly reduced the required RoM compared to RUD (*p* < 0.01) and combined PS‐RUD (*p* < 0.001), by 29.42% and 21.62%, respectively, and as shown in Figure 7c. The PS controller also reduced compensatory elbow flexion/extension movements compared to other controllers by 20.25% relative to RUD and 17.79% relative to combined PS‐RUD. This observation aligns with the findings from Peng et al. [17], where the book‐flipping task requiring rotational wrist movement exhibited significant reductions in compensatory joint angles when PS wrist control was applied. Given the kinematic similarity between book‐turning and doorknob rotation, our result supports the functional importance of aligning wrist DoFs with task demands. Without PS motion, rotating the door handle requires shoulder rotation, which in turn necessitates additional compensatory elbow movements to align the hand with the rotating handle. By enabling the wrist to rotate directly in the direction of the handle's rotation, the PS controller eliminates the need for these compensatory actions. It allowed for more efficient doorknob rotation and reduced unnecessary movement, though the difference was not statistically significant.

Similarly, for the screwdriver task, shoulder abduction/adduction shows a significant difference in RoM across the three controllers (*p* < 0.01). Comparison between controllers indicates that the PS controller significantly reduced the required RoM compared to RUD (*p* < 0.05) and combined PS‐RUD (*p* < 0.05) by 27.14% and 25.66%, respectively as shown in Figure 7d). Analogous to the doorknob task, the screwdriver task can also be categorized as a PS activity. The reduced motion at the shoulder also reduces the required compensatory motion at the elbow. Thus, the PS controller significantly reduced the compensatory motion at the elbow compared to that of RUD (*p* < 0.05), by 25.31%. A similar trend is observed between PS and combined PS‐RUD corresponding to a reduction of 23.95%, although it is not statistically significant. Furthermore, in the screwdriver task, a notable reduction in compensatory shoulder rotational motion (*p* < 0.01) was observed, particularly when using the PS controller (*p* < 0.05), by 47.70%. This reduction in compensatory shoulder rotation appeared only in the screwdriver task, not in the doorknob task, despite similar wrist motions. The difference arises from the distinct arm postures used in the last two tasks, which change the mechanical contribution of shoulder rotation to generating the required wrist movement. The doorknob task keeps the forearm nearly horizontal, requiring less shoulder involvement. In contrast, in the screwdriver task, the forearm is oriented downward (see Figure 4), making shoulder rotation more essential for task execution.

**FIGURE 8:**
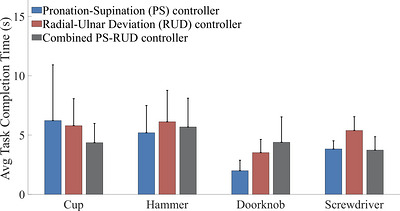
Task completion time across three wrist control strategies measured during four ADL tasks.

In addition to RoM, average task completion time was analysed to assess the functional performance of each controller (Figure 8). The combined PS‐RUD controller achieved the fastest times for the cup and screwdriver tasks, likely due to its dual‐DoF support, which allows a more natural wrist orientation. In the doorknob task, the PS controller showed the shortest completion time, aligning well with the task's rotational demands. This observation is consistent with prior findings by Bennett et al. [13], where an IMU‐based wrist controller reduced task completion time and compensatory torso movements during axial rotational tasks. While these trends were observed, no statistically significant differences in task completion time were found across controllers.

Interestingly, the RUD controller, despite being kinematically appropriate for the hammer task, showed the longest completion time. This may stem from the task's subtle need for PS adjustments, which the single‐DoF RUD controller could not accommodate. In contrast, the PS and combined PS‐RUD controllers may have facilitated better object alignment, thereby reducing the need for compensatory movements. These findings highlight the importance of adapting wrist DoFs to task‐specific motion requirements to enhance usability and functional efficiency in multi‐DoF prosthetic wrists.

### 4.2 User Feedback

**FIGURE 9:**
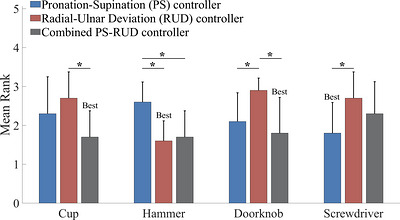
Mean preference ranking for performing four tasks with three different controllers. Smaller number indicates higher rank. *P < 0.05

**FIGURE 10:**
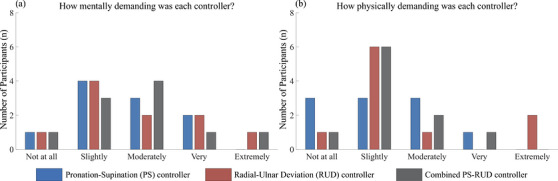
Participant feedback for (a) mental and (b) physical demands.

Participants evaluated each controller‐task combination based on preference, mental demand and physical demand. First, they ranked the three controllers (1 = most preferred, 3 = least preferred) for each task. The combined PS‐RUD controller was most preferred for the cup and doorknob tasks, the RUD controller for the hammering task and the PS controller for the screwdriver task (Figure 9). Friedman tests showed significant differences among all controllers for cup, hammer and doorknob tasks (*p* < 0.05), but for the screwdriver task, it wasn't significant. Post‐hoc Wilcoxon signed‐rank tests showed significant pairwise differences (*p* < 0.05) for the cup task (combined PS‐RUD vs. RUD), the hammer task (PS vs. RUD & combined PS‐RUD vs. PS), the doorknob task (PS vs. RUD & combined PS‐RUD vs. RUD) and the screwdriver task (PS vs. RUD). Participants also rated mental demand on a 5‐point scale. Most responses fell into the ‘slightly’ or ‘moderately’ demanding categories, with only two ‘extremely’ demanding responses across all conditions (Figure 10a). These results suggest that the cognitive load was generally manageable and may have been influenced by individual differences. Lastly, participants evaluated the physical demands of each controller using the same assessment criteria as for mental demands. Most of the participants rated RUD and combined PS‐RUD controller as ‘slightly’ physically demanding (See Figure 10b). However, there was no clear consensus for the PS controller, as some participants found it physically undemanding while others did not express a strong preference.

Comparing RoM analysis and user feedback, when the controller's supported motion closely matches the primary wrist movement required by the task, users exhibit fewer compensatory movements, greater satisfaction and reduced perceived physical effort. For RUD‐oriented tasks (cup and hammer), the RUD controller effectively reduced compensatory movements, while for PS‐based tasks (doorknob and screwdriver), the PS controller yielded similar benefits. Interestingly, in the cup and doorknob tasks, users preferred the combined PS‐RUD controller, which provides dual‐axis support, over single‐axis controllers. In the cup task, the combined PS‐RUD controller significantly reduced shoulder rotational movements compared to the RUD controller (*p* < 0.05), likely contributing to the lower reported physical demand. In the doorknob task, although RoM data indicated that the PS‐only controller minimized compensatory shoulder movements, participants still favoured the combined PS‐RUD controller, possibly due to the perceived benefits of dual‐axis control. Overall, participants tended to prefer single‐axis controllers for tasks requiring fine control (e.g., screwdriver, hammer), as they offer more direct and intuitive manipulation. In contrast, for lower‐precision tasks, the combined PS‐RUD controller was favoured for its ability to reduce compensatory movements across multiple axes.

### 4.3 Limitation and Future Studies

Several limitations were observed during real‐time evaluation of the multi‐DoF wrist controller. First, participants exhibited a noticeable learning curve during pre‐experiment training as they adapted to the VR tasks. In the future, a more rigorous participant training protocol will be implemented to minimize adaptation time and improve training efficiency for the VR tasks. Second, this study was conducted with non‐disabled participants, which limits direct generalization to individuals with limb loss. Individuals with upper‐limb amputations may exhibit distinct residual‐limb kinematics, socket‐related constraints and compensatory movement strategies, which may influence controller intuitiveness, task performance, perceived effort and learning effects. Future studies will include individuals with upper‐limb amputations to directly assess usability, compensatory movement patterns and overall controller performance. Third, because the primary interest was quantifying compensatory movements, RoM was the only kinematic metric analysed. In the future, additional measures such as joint velocity and trajectory smoothness will be examined to characterize movement quality. Fourth, the VR environment limits evaluation of the controller because it does not capture the mechanical and interaction constraints of a physical prosthetic device. Future work will include evaluation on a physical prosthetic wrist developed in our lab [23]. Therefore, the findings should be interpreted as preliminary observations within a controlled participant group. Future work will include a larger sample size to obtain more stable effect estimates and to improve generalizability. Sixth, the wrist brace removed active wrist motion to simulate the absence of wrist mobility, however, this does not fully reproduce the movement strategies or compensatory behaviours of amputee users. Therefore, the findings should be interpreted as an initial evaluation rather than a definitive representation of amputee performance. Last, although our model was selected for its low computational cost and demonstrated real‐time performance, recent models, such as recurrent or convolutional models, were not explored. In future work, we will evaluate these models and incorporate multi‐modal sensors, including sensor fusion that combines sEMG signals with IMU‐based motion synergies and other complementary sensing modalities, to improve user intent estimation and controller accuracy [24]. Future work will also aim to expand active wrist functionality to three degrees of freedom (3‐DoF) to accommodate a broader range of ADLs.

## 5 Conclusion

This study evaluated an IMU‐based prosthetic wrist control framework that leverages upper‐limb movement synergies, employing a neural network classifier and random forest regression to infer user intent in real time and control multi‐DoF wrist motion within a virtual physics engine. The system was tested across three controller types and four representative ADL tasks. Results suggest that synergy‐based, IMU‐driven control may support the intuitiveness and efficiency of prosthetic wrist use. The findings also suggest that adapting control strategies to task‐specific demands may improve usability by reducing compensatory movements in selected task‐controller combinations, although task completion time differences were not statistically significant. Participant feedback further suggested that these controller configurations may be associated with lower perceived physical and cognitive effort under the tested conditions. Future work will focus on validating these findings with a physical prosthesis and further refining the control algorithms to improve adaptability across a wider range of ADLs.

## Author Contributions

**Deepti Bharadwaj**: investigation, data curation, formal analysis, writing – original draft. **Ingyun Ahn**: formal analysis, writing – review & editing. **Saurabh S. Deshmukh**: formal analysis, validation, writing – original draft. **Jiyeon Kang**: conceptualization, supervision, writing – review & editing.

## Funding

Ministry of Health and Welfare under Grant RS‐2024‐00417629; by the National Research Foundation of Korea (NRF) funded by the Korean government (MSIT) under Grant Nos. RS‐2026‐25483614 and RS‐2024‐00361688 (Bio & Medical Technology Development Program); and by the Institute of Information & Communications Technology Planning & Evaluation (IITP) grant funded by the Korean government (MSIT) (No. 2019‐0‐01842, Artificial Intelligence Graduate School Program, GIST).

## Conflicts of Interest

The authors declare no conflicts of interest.

## Data Availability

The data that support the findings of this study are available from the corresponding author upon reasonable request.
